# Neural Correlates of Attachment Representation in Patients With Borderline Personality Disorder Using a Personalized Functional Magnet Resonance Imaging Task

**DOI:** 10.3389/fnhum.2022.810417

**Published:** 2022-02-24

**Authors:** Dorothee Bernheim, Anna Buchheim, Martin Domin, Renate Mentel, Martin Lotze

**Affiliations:** ^1^Department of Psychiatry and Psychotherapy, University Hospital of Greifswald, Greifswald, Germany; ^2^Department of Child and Adolescent Psychiatry and Psychotherapy, University Hospital of Ulm, Ulm, Germany; ^3^Department of Psychology, University of Innsbruck, Innsbruck, Austria; ^4^Functional Imaging Unit, Department of Diagnostic Radiology and Neuroradiology, University of Greifswald, Greifswald, Germany

**Keywords:** neural correlates of attachment representation borderline personality disorder, adult attachment representation, fear of abandonment, aloneness, social pain, anterior medial cingulate cortex, hypermentalization

## Abstract

**Background:**

Fear of abandonment and aloneness play a key role in the clinical understanding interpersonal and attachment-specific problems in patients with borderline personality disorder (BPD) and has been investigated in previous functional Magnet Resonance Imaging (fMRI) studies. The aim of the present study was to examine how different aspects of attachment representations are processed in BPD, by using for the first time an fMRI attachment paradigm including personalized core sentences from the participants’ own attachment stories. We hypothesized that BPD patients would show increased functional involvement of limbic brain regions associated with fear and pain (e.g., the amygdala and the anterior cingulate cortex) when presented personalized attachment relevant stimuli representing loneliness compared to healthy controls (HC).

**Methods:**

We examined the attachment classifications of 26 female BPD patients and 26 female HC using the Adult Attachment Projective Picture System (AAP). We used an fMRI-adapted attachment paradigm to investigate the neural correlates of attachment. All participants were presented three personalized (vs. neutral) sentences extracted from their AAP attachment narrative, combined with standardized AAP pictures representing being alone (monadic) or in interactive (dyadic) attachment situations.

**Results:**

As expected, the classification of unresolved attachment was significantly greater in BPD compared to HC. BPD patients showed increased fMRI-activation in brain areas associated with fear, pain, and hyperarousal than HC when presented with personalized attachment-relevant alone stimuli. In particular, pictures with monadic attachment situations induced greater anterior medial cingulate cortex, anterior insula, amygdala, thalamus and superior temporal gyrus activation in the patient group.

**Conclusion:**

The results point to increased fMRI-activation in areas processing emotional distress and painful experiences in BPD patients. In particular, the emotional cascade reflecting attachment distress was evoked by combining monadic pictures, representing abandonment and aloneness, with the patients’ personalized narrative material. Our results confirmed and replicated previous results that illustrate once again the high relevance of aloneness and feelings of abandonment for BPD in the context of attachment trauma. Moreover, our results support the hypothesis of hypermentalization in response to attachment distress as a core feature of social-cognitive impairment in BPD associated with common treatment implications across different therapeutic orientations.

## Introduction

Borderline personality disorder (BPD) is characterized by affect dysregulation, behavioral dyscontrol, and interpersonal hypersensitivity with developmental roots in insecure infant–parent attachment and adverse childhood experiences (Leichsenring et al., 2011). Epidemiological studies (Zanarini, 2000) demonstrate that sexual abuse and emotional neglect are overrepresented in borderline patients. According to the DSM-5, interpersonal dysfunction in BPD is characterized by an anxious preoccupation with real or imagined abandonment, and disturbed emotion processing and social interaction have been a focus of a large number of BPD studies (Schmahl et al., 2014). For the first time, Gunderson (1996) proposed associations between BPD patients’ specific fearful inability to be alone and dysregulated attachment behavior in current relationships with childhood neglect and abandonment. Several studies identified insecure attachment, mostly insecure-preoccupied and unresolved attachment patterns as predominant in BPD (Fonagy et al., 1996; Agrawal et al., 2004; Levy, 2005; Bakermans-Kranenburg and van IJzendoorn, 2009; Beck et al., 2017; Buchheim and Diamond, 2018). We may conclude that childhood attachment-related trauma plays a central role in the pathogenesis of BPD (e.g., Keinänen et al., 2012; Herpertz and Bertsch, 2015).

Attachment theory posits that the state of “being left alone or abandoned” is one of the most frightening and emotionally painful experiences for humans and being alone activates the attachment system in children and adults on behavioral, representational and neurobiological levels (Buchheim et al., 2008; Gander and Buchheim, 2015; Spitoni et al., 2020). The “internal working model of attachment” (Bowlby, 1969; Bretherton and Munholland, 2008) is conceived as a mental representation of attachment built from early experiences with caregivers ranging from sensitive care and protection to abandonment, abuse and loss. These representational attachment elements and can be evaluated using interview measures like the Adult Attachment Projective Picture System (AAP) (George and West, 2001, 2012). The AAP is a reliable and valid instrument used to assess the adult attachment representation. Classifications include organized patterns (secure, insecure-dismissing, insecure-preoccupied) and a “disorganized” attachment pattern termed “unresolved” for trauma.

The biographical background of BPD patients often includes interpersonal attachment trauma such as violence or neglect; therefore, it is not surprising that a consistently high percentage of patients are judged as “unresolved” (Agrawal et al., 2004; Keinänen et al., 2012; Buchheim and Diamond, 2018). This pattern is characterized by the inability to mobilize an “internalized secure base of attachment” when attachment is activated and is associated with severe emotional dysregulation and psychopathology (Buchheim et al., 2017a). Moreover, one previous study reported that AAP attachment narratives of BPD patients included high numbers of trauma features, like “helplessness” or “suicide,” especially in response to the alone picture stimuli (Buchheim et al., 2008; Buchheim and George, 2011).

Neural processes linked to human attachment (Buchheim et al., 2017b) and social exclusion have been repeatedly studied in functional magnet resonance imaging (fMRI) studies of healthy adults. Most typically, these studies experimentally simulated social exclusion (“Cyber-Ball”) during scanning, which was linked to the experience of “social pain” (Vijayakumar et al., 2017). Social pain during social exclusion was evidenced in healthy control subjects (HCs) with neural activity in the anterior midcingulate cortex (aMC; which is actually the same area as the dorsal anterior cingulate cortex dACC) associated with increased distress after social exclusion. By comparison, activity in the right ventral prefrontal cortex (vPFC) was associated after social exclusion with diminished distress in HC (Eisenberger et al., 2003). Attachment style questionnaire fMRI studies also demonstrated a relationship between attachment anxiety and increased neural dACC/aMCC activity (Gillath et al., 2005; DeWall et al., 2012).

Buchheim et al. (2006) first investigated the neural signature of attachment representations in HCs using an fMRI-adapted version of the AAP. Their analysis of the AAP narratives showed that only *unresolved* HCs (vs. organized) showed increases in activity in the right inferior frontal cortex, left superior temporal sulcus (STS), left head of the caudate nucleus, and bilateral temporal lobe (left amygdala-hippocampus area, right amygdala). In a following study, BPD patients were compared with HCs using the same paradigm. The results showed, that only the patients with *unresolved* (vs. organized) attachment representation showed increased neural activity in the aMCC related to the monadic pictures. The findings also showed increased neural activity in the right STS as well as decreased neural activity in the right parahippocampal gyrus related to dyadic pictures (Buchheim et al., 2008).

Moreover, increased dACC (aMCC) activity among BPD patients compared to HCs was found in the Cyber-Ball paradigm and increased activation of the dorsolateral- and dorsomedial prefrontal cortex (dlPFC, dmPFC), precuneus, and anterior insula (Domsalla et al., 2014). This result pattern has been interpreted as a tendency for BPD patients to engage in over-interpretation and over-attribution (“hypermentalization”) oblivious to the intention of others (Sharp et al., 2015; Somma et al., 2019). By contrast, Schmahl et al. (2003) found increased neural activity in the bilateral dlPFC (middle frontal gyrus, Brodmann’s areas 9/10) and right cuneus but *decreased* neural activity in the right anterior cingulate cortex (Brodmann’s areas 24/32) in women with BPD, using personalized scripts of abandonment memories in an fMRI environment.

To summarize the previous findings, the following brain areas (“attachment network”) appear to be involved in attachment relevant tasks: (1) mentalization-related processes and self-awareness: posterior superior temporal sulcus (pSTS), medial prefrontal cortex (mPFC), and temporal poles, (2) processes of perception/empathy of pain and fear: aMCC, amygdala, anterior insula, and (3) processes involved in conflict monitoring, cognitive control, and reaction inhibition: aMCC, vPFC, and dlPFC (Labek et al., 2016; Sosic-Vasic et al., 2019).

The aim of the present study was to examine how different aspects of attachment representations are processed in BPD, by using for the first time an fMRI attachment paradigm including personalized core sentences from the participants’ own attachment stories. We assumed the following hypotheses: First, based on previous work (Buchheim et al., 2008) and recently-published behavioral results from our sample (Bernheim et al., 2018, 2019), we expected stronger brain activations in attachment-associated areas among BPD patients compared to HC, especially when presented monadic (vs. dyadic) pictures of the AAP, specifically localized in the aMCC (ROI). Second, according to the previous work (Buchheim et al., 2012), we hypothesized stronger fMRI-activations of the above mentioned “attachment network” among BPD patients when confronted with personalized narrative material vs. neutral sentences in the context of the monadic AAP pictures. Third, we assumed an association between brain-activations of the “attachment network” and the participants’ attachment dysregulation, rated by the number of “unresolved” AAP stories.

## Materials and Methods

### Procedure

This study is part of a larger fMRI study of BPD patients treated with outpatient Dialectical Behavioral Therapy (DBT). Subject characteristics, inclusion and exclusion criteria, and diagnostic assessments were more in detail described in a publication focusing on the behavioral data only (Bernheim et al., 2019). The study was approved by the Ethical Commission of the University of Greifswald (BB 136/10). The study conforms to recognized standards of the Declaration of Helsinki. All persons gave their informed consent prior to inclusion.

### Sample

The participants were 26 female right-handed BPD patients and 26 female right-handed healthy controls (HC) matched for age, and education. The patients were recruited at psychiatric hospitals in Greifswald, Germany, and the HC via advertisements using university and community platforms. All participants were examined using the Structured Clinical Interview for DSM-IV, axis II (SKID-II; inclusion criteria: cut-off ≥ 5 points; Wittchen et al., 1997), Borderline Personality Inventory (BPI; Leichsenring, 1997) and Borderline Symptom List-23 (BSL-23; Bohus et al., 2001). Patients with legal guardianship, mental retardation (IQ < 70) or florid psychotic symptoms were excluded. Only HC participants willing to receive psychiatric assessments and attachment interviews were included. HC individuals with current or anamnestic psychiatric disorders, a history of psychotherapeutic treatment or psychiatric medications were excluded. Individuals who showed contraindications to fMRI procedures or who were pregnant were excluded. While antidepressant medications are probably unavoidable in a BPD sample, antipsychotic medication strongly alters brain activity. For this reason, nine of 26 patients (34.6%) took selective serotonin reuptake inhibitors (SSRI’s), but none of the patients received a neuroleptic medication. Medication dosage was kept at minimum throughout the study.

### Measurements

#### Clinical Instruments

The following well-established diagnostic assessments were applied to investigate axis I diagnoses (DSM-IV), trauma history, level of crystalline intelligence and global symptom severity in both groups: Munich - Composite International Diagnostic Interview (M-CIDI/DIA-X; Wittchen et al., 1996); Posttraumatic Diagnostic Scale (PDS; Ehlers et al., 1996); Multiple-Choice Vocabulary Test, version B (Lehrl, 1995); Brief Symptom Inventory (BSI-53; Franke, 2000).

The attachment classifications were judged using the AAP (George and West, 2001, 2012). The coding and classification procedure is described more in detail below. In addition to the classifications, we counted the number of individual AAP stories that were designated as “unresolved”. All participants were presented with personalized AAP core sentences from their own narratives in the fMRI procedure and asked to rate them after scanning.

#### Attachment Measure as the Basis for the Functional Magnet Resonance Imaging Paradigm

Participants were administered with the fMRI-adapted version of the AAP (Buchheim et al., 2012). The AAP (George and West, 2001, 2012) is a well-validated interview measure that assesses adult attachment mental representation. This measure was used in two ways. The first was to determine participants’ attachment classifications and use the narratives to develop individualized individual narratives. The second was to use this material in the experimental fMRI paradigm. The AAP is based on the analysis of “story” responses to a set of theoretically-derived attachment-related drawings of scenes depicting solitude, illness, separation, death and potential maltreatment. Drawings portray adults and children alone (three monadic pictures, representing abandonment) as well as adult-adult/adult-child dyads (four dyadic pictures, representing interpersonal distress) and one neutral picture. Individuals are asked to tell a story to each picture following a standardized set of interview probes. The classification is derived by evaluating the response patterns for the whole set of seven picture stimuli, each response of which is evaluated for content, discourse, and defensive processes. Organized attachment is defined in the AAP, following the attachment literature at large, as secure, and insecure-dismissing and preoccupied classifications; any frightening or threatening material that appears in the story is contained (e.g., desperately alone, death, attack, and abuse – note that not all stories contain this material). Transcripts are judged unresolved when there is no evidence of representational containment of frightening elements in at least one story. The nuances of coding are beyond the scope of this manuscript, and the reader is referred to George and West (2012) for details. AAPs are transcribed verbatim from audio recordings for analysis.

The AAP has demonstrated solid psychometric properties, including test-retest reliability, inter-judge reliability, and convergent and discriminant validity (George and West, 2001, 2012; Buchheim and George, 2011; Buchheim et al., 2018). AAP classification in the present study was performed by two independent certified judges. Inter-rater reliability showed significant concordance for the four-group classification (κ = 0.95, 95%-confidence interval [0.88, 1.04], *p* < 0.001), and for the two-group classification (organized vs. unresolved, κ = 0.96), 95%-confidence interval [0.91, 1.00], *p* < 0.001. Both independent and blind raters agreed in 50 out of 52 cases (Bernheim et al., 2018).

Statistical evaluation of rating data was performed with SPSS (IBM coop.) version 21. We used chi square tests to compare ratings between samples (non-parametric testing needed because normal distribution of ratings cannot be expected).

### Functional Magnet Resonance Imaging Task

#### Personalized Core Sentences

Three personalized sentences were extracted per picture for each participant following the procedure described by Buchheim et al. (2012). Sentences included the major core elements of the stories: (1) event description, e.g., “*This is a lonely child, cut off from the world*,” (2) thoughts/feelings, e.g., “*Nobody plays with her, she feels desperate*,” and (3) story outcome, e.g., “*She is helpless without any hope*.” As a contrast for the fMRI setting, three non-personalized, neutral sentences were used per AAP picture, being identical for all participants and describing the environment of the picture only (e.g., “*two curtains on the left and on the right of the window;”* see Supplementary Material 1, note: AAP example picture 2: “Window”).

#### Functional Magnet Resonance Imaging Design

**FIGURE 1:**
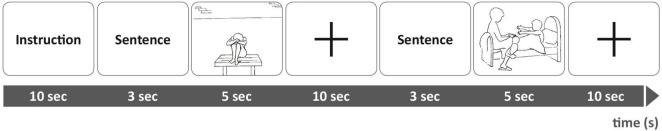
Experimental setting during functional magnet resonance imaging (fMRI). *Top line*: Order of stimuli presentation during scanning. *Bottom line*: Duration of presentation in seconds.

The task started with the instruction to carefully read the sentences and look at the pictures attentively. Each trial had the following order of presentation: sentence - picture – fixation cross (see Figure 1).

The individualized core sentences were paired to the respective AAP picture to constitute “personal” trials tailored to each participant. The same pictures were also paired to neutral sentences that described only the situational environmental elements depicted in the picture. These pairings were identical for all participants. The AAP picture stimuli are always administered in a designated order (George and West, 2012). We, therefore, presented the stimuli of each condition in sets comprising seven consecutive trials of the same condition, interleaved by 10 s fixation periods. In total, we presented six sets of personalized trials (i.e., in total 42 trials), and six sets of neutral trials, in an alternating fashion. Three sets contained seven personalized sentence-picture-combinations and three sets contained seven neutral sentence-picture-combinations, equal for all participants. These sets were presented alternating between sets with personalized stimuli and sets with neutral textual stimuli. In total, there were 84 trials with a total duration of 25 min and sets of trials were presented in immediate succession with no periods of rest interleaved.

Participants were asked to rate autobiographic and emotional relevance for each of the personalized sentences after the fMRI measurement using a questionnaire (Buchheim et al., 2012; Bernheim et al., 2018).

### Training Procedures

Before the fMRI experiment, all participants were informed that they would be presented with several sentences, combined with the AAP pictures. All participants were instructed not to move their head or body during fMRI and to focus on the sentences and pictures presented.

### Image Acquisition

A 3T Siemens Magnetom Verio (Erlangen, Germany) with 32-channel head coil was used to acquire a T1 whole head volume for structural mapping, T2*-weighted echo-planar images (EPIs) for functional mapping, and gradient echo for unwarping of the EPIs.

Echo-planar images were characterized by a repetition time (TR) of 2,000 ms, echo time (TE) of 23 ms, flip angle α of 90°, and Field Of View (FOV) of 208 mm. Each volume consisted of 33 slices (transversal; AC-PC aligned with additional 20° to minimize susceptibility artifacts in the frontobase) with voxel size of 2 mm × 2 mm × 3 mm and spacing between slices of 1 mm. For each participant, 756 whole head EPI volumes were obtained, the first two dummy volumes in each session being automatically discarded to allow for T1 equilibration effect. 34 phase and magnitude images were acquired in the same FOV by a gradient echo (GRE) sequence with TR = 488 ms, TE(1) = 4.92 ms, TE(2) = 7.38 ms, and α = 60° to calculate a field map aiming at correcting geometric distortions in EPI images. The T1-weighted three-dimensional image (MPRAGE) was used as spatial high-resolution structural image. The total number of sagittal anatomical images/slices was 176 (*R* = 1,900 ms, TE = 2.52 ms, α = 90°, voxel size 1 mm × 1 mm × 1 mm, matrix size = 256 mm × 256 mm).

### Functional Magnet Resonance Imaging Data Analysis

Data was analyzed using SPM12 (Wellcome Department of Cognitive Neuroscience) implemented in MATLAB (MathWorks, Inc., Natick, MA, United States). Unwarping of geometrically distorted EPIs was performed in the phase encoding direction using the FieldMap Toolbox. Each time-series was realigned to the first image of each session and re-sliced. EPIs were co-registered to the T1-weighted anatomical image, and T1-weighted images were segmented to localize gray and white matter and cerebrospinal fluid. This segmentation was the basis for spatial normalization to the Montreal Neurological Institute (MNI) template using the DARTEL approach of SPM. Here, a group template was calculated, being refined iteratively by diffeomorphic registration steps, co-registered to the SPM MNI template, and used to spatially normalize the functional images while applying a Gaussian Kernel smoothing filter (9 mm × 9 mm × 9 mm full-width at half maximum) to improve spatial alignment and increase the signal-to-noise-ratio. Six movement parameters estimated during the realignment procedure were introduced into the model as covariates to control for variance due to head displacements. A temporal high-pass filter (128 s) was applied to remove slow signal drifts. Individual statistical maps for main effects (personalized monadic pictures/personalized dyadic pictures) and contrasts (personalized monadic pictures minus personalized dyadic pictures; monadic pictures + personalized minus neutral sentences) were calculated using the general linear model. First level contrast images of each subject were used for group statistics calculated as a random effect analysis at the second level. A one-sample *t*-test was performed to assign significant activations. A two-sample *t*-test was accomplished at the second level for between subject groups. A linear regression was calculated for the patients *U*-scores and fMRI-activation during monadic picture presentation, with personalized minus neutral sentences.

Anatomical classification of fMRI-activation (MNI-space) was performed using Anatomy Toolbox Version 1.7 (Eickhoff et al., 2005) and with Anatomy with Automated Anatomical Labeling (AAL) (Tzourio-Mazoyer et al., 2002). Differentiation of the cingulate cortex (AAL-mask) was performed by defining the MCC as the most rostral (*z* > 30). We corrected for the whole brain volume (*p* < 0.05, familywise error rate; FWE) by calculating main effects within each group. We used a region of interest (ROI) approach and corrected for false positive results with *p*_*FWE*_ < 0.05 corrected for ROI-volume for calculating interactions and differences between groups. Following the core studies leading to our hypotheses, these were comprised of (1) STS, mPFC, temporal poles, (2) aMCC, amygdala, anterior insula, and (3) vmPFC and dlPFC. Linear regression analysis was used to evaluate our expected association of aMCC-activation (ROI-approach; *p*_*FWE*_ < 0.05) based on the number of unresolved rated AAP pictures in BPD patients.

## Results

### Sample Characteristics and Symptom Severity

Participants in the two groups did not significantly differ regarding age [BPD, *M* = 26.45, *SD* = 7.04; HC, *M* = 26.80, *SD* = 6.58; *t*(50) = 0.19, *p* > 0.05], intellectual performance/IQ [BPD, *M* = 108.81, *SD* = 14.65; HC, *M* = 107.96, *SD* = 9.49; *t*(50) = −0.25, *p* > 0.05], and educational background (BPD, 53.8%, *n* = 14; HC, 61.5%, *n* = 16; χ^2^ = 2.13, *p* = 0.13). BPD patients scored significantly higher than HC participants in the borderline and global symptom severity and exhibited a comorbidity of five additional axis I diagnoses and one additional axis II diagnosis (DSM-IV) (see Table 1).

**TABLE 1:** Symptom severity of patients with Borderline Personality Disorder (BPD) and Healthy Control Subjects (HC).

Variable	BPD (*n* = 26)	HC (*n* = 26)		Z^a^/Chi^2^	*p* two-tailed
**Symptom severity**					
Global symptom severity (BSI-GSI) (M/SD)	1.64/0.67 38.17^a^	0.15	0.13 13.34^a^	8.50^a^	<0.001
Number of trauma (PDS) (M/SD)	2.62/1.44	0.77	1.14	−5.11	<0.001
Posttraumatic stress disorder (DIA-X) (%/*n*)	38.5/10	0.0	0		
**Number of diagnoses (M/SD)**					
• Axis I (DSM-IV)	4.50/1.08	0.00	0.00		
• Axis II (DSM-IV)	2.85/1.20	0.00	0.00		

*^a^Mann-Whitney U-test and rank-sum.*

### Attachment Results

As expected, both groups differed significantly in their distribution of organized versus unresolved attachment representations. BPD patients showed 42.3% organized and 57.5% unresolved classifications, while healthy controls showed 84.6% organized and 15.4% unresolved classifications [χ^2^(3) = 14.73, *p* = 0.002]. BPD patients with organized versus unresolved attachment representations did not differ significantly concerning age or borderline/global symptom severity.

Analyzing the amount of number of individual AAP stories, that were designated as unresolved, the *Between-* group comparisons showed that BPD patients showed a significantly higher frequency of unresolved monadic stories than HCs (see Table 2). *Within-group* comparisons showed that BPD patients showed significantly more unresolved stories for monadic as compared with dyadic stimuli, respectively *M* = 0.69, *SD* = 0.74 and *M* = 0.15, *SD* = 0.37 [*t*(25) = 3.61, *p* < 0.01].

**TABLE 2:** Number of “unresolved” rated narratives related to the Adult Attachment Projective Picture System (AAP) pictures in the AAP interview (BPD vs. HC).

Variable	BPD (*n* = 26)		HC (*n* = 26)			*p* two-tailed
*U*-Scores (U^b^) in AAP narratives						

**AAP Pictures**	**%/*n***	***U***	**%/*n***	***U***	**Z^a^**	***p***

• Monadic	53.9/14	18	15.3/5	5	2.857	0.004
• Dyadic	15.4/4	4	100/26	0	2.062	0.039

*^a^Mann-Whitney U-test and rank-sum.*

*^b^U-Scores: number (n) of “unresolved” rated AAP narratives related to the AAP pictures.*

As mentioned above all participants were asked to rate the personalized AAP core sentences from their own narratives after scanning. BPD patients rated the personalized sentences for all AAP pictures as significantly higher in autobiographical relevance [*t*(50) = 4.14, *p* < 0.001] and negative emotional valence [*t*(50) = 2.83, *p* < 0.01] than HC.

### Imaging Results

The major goal of our fMRI study was to differentiate between personalized and neutral narrative material combined with attachment pictures representing alone or dyadic situations in BDP patients versus healthy controls. We first analyzed the main effects of all AAP pictures and personalized sentences and then compared monadic versus dyadic trials for each group.

#### Effects of the Task in Both Groups

A main effects analysis of all AAP pictures and personalized sentences participants in both groups showed bilateral frontotemporal and occipital activation pattern (see Supplementary Material 2).

#### Specific Effects Observed in Borderline Personality Disorder Compared to Healthy Control

##### Monadic Adult Attachment Projective Pictures and Personalized Sentences

**FIGURE 2:**
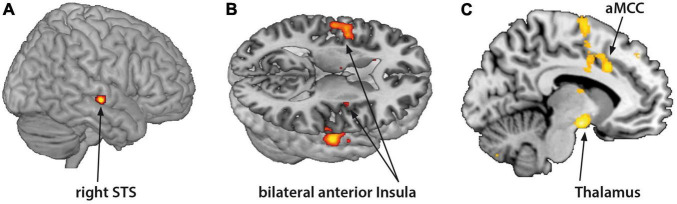
Functional magnet resonance imaging-activation during observation of monadic Adult Attachment Projective (AAP) pictures with personalized sentences (BPD minus HC). The borderline personality disorder (BPD) patients showed increased fMRI-activation in the **(A)** right superior temporal sulcus (STS), **(B)** bilateral anterior insula, **(C)** anterior medial cingulate cortex (aMCC) and thalamus (*p* < 0.05, FWE ROI-corrected).

Borderline personality disorder patients showed increased fMRI-activation in the bilateral anterior insula, right STS, aMCC, and thalamus when confronted with monadic AAP pictures combined with personalized sentences (see Figure 2 and Table 3).

**TABLE 3:** Monadic AAP pictures and personalized sentences (BPD minus HC).

Area	*t*-Value	Cluster	FWE corrected	*x*	*y*	*z*
Right STS	4.22	105	0.01	56	−19	−5
Anterior medial cingulate cortex	4.07	389	0.03	−8	18	34
Left thalamus	3.56	8	0.03	−8	−7	15
Left anterior insula	3.99	42	0.01	−42	0	6
Right anterior insula	3.41	25	0.04*	34	0	12

*Cluster-level threshold p < 0.05 FWE corrected.

##### Monadic Minus Dyadic Adult Attachment Projective Pictures and Personalized Sentences

**FIGURE 3:**
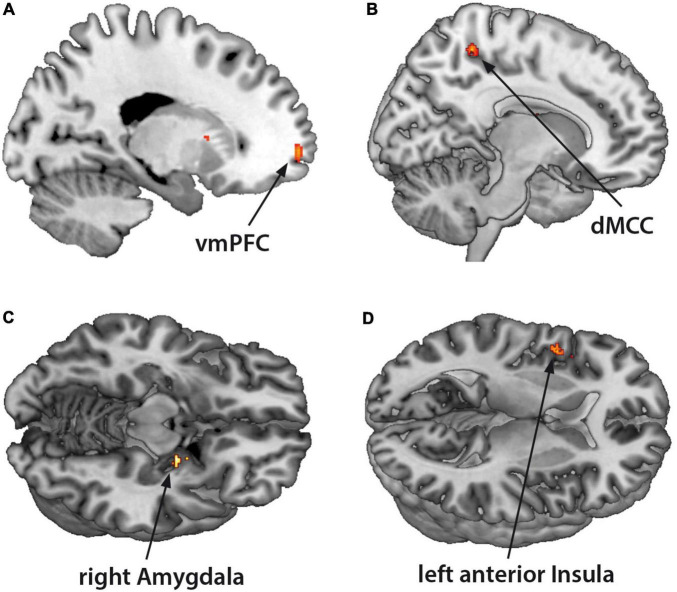
Functional magnet resonance imaging-activation during observation of monadic minus dyadic AAP pictures with personalized sentences (BPD minus HC). The BPD patients showed increased fMRI-activation in the **(A)** ventromedial prefrontal cortex (vmPFC), **(B)** dorsal medial cingulate cortex (dMCC), **(C)** right amygdala, and **(D)** left anterior insula (*p* < 0.05, FWE ROI-corrected).

The fMRI-activation did not differ between the two groups when confronted with dyadic AAP pictures and personalized sentences. Contrasting monadic minus dyadic pictures with personalized sentences, the BPD patients showed increased fMRI-activation in the left vmPFC, dMCC, left anterior insula, and right amygdala (see Figure 3 and Table 4).

**TABLE 4:** Monadic minus dyadic AAP pictures with personalized sentences (BPD minus HC).

Area	*t*-Value	Cluster	FWE corrected	*x*	*y*	*z*
Left vmPFC	3.84	35	0.03	−18	62	−2
Left anterior insula	3.75	57	0.02	−40	20	−8
MCC/PCC	3.97	56	0.04	12	−42	54
Right amygdala	3.65	16	0.02	22	−6	−12

##### Monadic Adult Attachment Projective Pictures and Personalized Minus Neutral Sentences

Borderline personality disorder patients as compared with the HC group showed a higher fMRI-activation in the right amygdala [MNI-coordinates (*x*, *y*, *z*): 21, −4, −11; *t* = 3.81; p_*FWE*_ = 0.032, 46 voxel], when presented monadic AAP pictures and personalized minus neutral sentences.

#### Additional Results

##### Linear Regression for Functional Magnet Resonance Imaging-Activation During Observation of Critical Monadic Picture Material and the Scores of Unresolved Attachment (BPD)

We found increased fMRI-activation in the BPD group in the aMCC in linear correlation to the number of “unresolved” rated AAP pictures (“*U*-scores”) per AAP narrative when patients were confronted with monadic AAP pictures and personalized minus neutral sentences (coordinates: −6, 33, 33; *t* = 5.44; *p*_*FWE*_ = 0.022; 43 voxel).

## Discussion

### Attachment Results

The present study explored the neural signature of attachment representations in BPD patients (BPD) compared to healthy control subjects (HCs), using the Adult Attachment Projective Picture System (AAP; George and West, 2001, 2012) using an adapted fMRI procedure (Buchheim et al., 2012).

The attachment results of the current study (Bernheim et al., 2018, 2019) were consistent with previous findings (Fonagy et al., 1996; Agrawal et al., 2004; Buchheim et al., 2016, 2017a; Buchheim and Diamond, 2018). BPD patients had significantly greater proportion of unresolved attachment classifications than HC. Similarly, they showed a significantly greater number of traumatic fear indicators (stories coded as unresolved) in their responses to monadic pictures. Following attachment theory, the monadic AAP pictures activate attachment in the representational the state of being alone (Buchheim and George, 2011). Our results confirmed previous results that illustrate once again the high relevance of aloneness and feelings of abandonment for BPD (Schmahl et al., 2003, 2014; Buchheim et al., 2008). However, we have to mention that emotional neglect, trauma, and attachment issues contribute to the development of borderline pathology within a broad multifactorial etiological model (Leichsenring et al., 2011; Mosquera et al., 2014).

### Functional Magnet Resonance Imaging Results

#### Monadic Attachment Stimuli

The fMRI-activation of BPD patients corresponded to our behavioral results showing differences between responses to the *monadic* pictures combined with personalized sentences and the HC. Consistent with Buchheim et al. (2008), the BPD patients showed higher fMRI-activation in the aMCC, and higher fMRI-activation in the bilateral anterior insula, right STS, and thalamus. Several studies have investigated the involvement of MCC in pain processing (Apkarian et al., 2005; Vogt, 2005; Taylor et al., 2009). The aMCC and anterior insula are both involved in examining empathy responses to physical pain in others (Lotze et al., 2007; Lamm et al., 2011). Taylor et al. (2009) postulated a network consisting of aMCC and anterior insula integrating information of emotional salience and responsible for the formation of a subjective, physical representation of pain. This finding supported the roles of the aMCC and insula as key relay sites during neural processing of pain (Wilcox et al., 2015).

Conversely, the posterior superior temporal sulcus (pSTS) in the temporoparietal junction (and the mPFC) has been described in relation to tasks measuring empathy for social pain (exclusion, shame) of other persons and assigned to the “mentalization network” (Frith and Frith, 2003). In this regard, the embarrassment on behalf of others is a kind of “social pain” that engages the temporal pole and the mPFC (central structures of the mentalizing network) together with the anterior insula and anterior cingulate cortex (Paulus et al., 2015). Interestingly, *sharing* others’ embarrassment in this paradigm additionally stimulated the pSTS, which exhibited increased functional integration with inferior parietal and insular cortex areas. These findings, *inter alia* characterize the unique role of pSTS in sharing others’ affective state (Paulus et al., 2015).

The BPD group in the current study especially showed increased fMRI-activation of the thalamus while viewing monadic AAP pictures with personalized sentences. Previous studies presenting validated pictures from the International Affective Picture System (Lang et al., 2008) in an fMRI design combined with psychophysiological measures, highlighted the role of the thalamus in the arousal dimension of emotional processing (Anders et al., 2004). According to Vogt (2005), activation of the circuit by nociceptive inputs from the thalamus induces fear and memories of similar events and triggers outcome prediction. We suggest that increased thalamic activation in the BPD group might be associated with increased arousal during monadic picture presentation with personalized attachment material due to the fact that BPD patients demonstrated especially high number of traumatic fear indicators in the responses to the monadic AAP pictures.

#### Monadic Minus Dyadic Attachment Stimuli

In contrast to previous results (Buchheim et al., 2008), we did not observe differences in neural processing between BPD patients and the HC while viewing dyadic AAP pictures. George and West (2012) developed the dyadic attachment pictures to portray scenes of attachment-caregiving interaction, which implicates *potential* social rejection. By contrast, George and West (2012) developed the monadic attachment pictures to portray aloneness, which is likely to activate feelings of abandonment. It follows that the feeling of abandonment evoked by monadic AAP pictures would show a stronger impact on fMRI-activation of the attachment network. This hypothesis would be untangled by contrasting monadic minus dyadic AAP pictures and related personalized sentences in both groups. Our results showed that BPD patients exhibited increased fMRI-activation in the dorsal part of MCC and the left anterior insula in response to monadic AAP pictures, accompanied by a neural hyperactivation of the right amygdala and vmPFC. Brain imaging studies suggest that BPD patients show structural and functional alterations in a frontolimbic network, in particular reduced amygdala volume and enhanced functional bilateral amygdala activation responding to emotional stimuli such as fearful scenes and facial expressions (Donegan et al., 2003; Minzenberg et al., 2007; Domes et al., 2009; Buchheim et al., 2016). The ventromedial prefrontal cortex (vmPFC) is a key neural substrate of human social cognitive and affective function and serves to regulate negative affect via top-down inhibition of brain regions involved in negative emotions, in particular the amygdala. Kamphausen et al. (2013) used an instructed fear task combined with fMRI and skin conductance response (SCR) and, *inter alia*, found increased connectivity of the amygdala with vmPFC in BPD patients compared to HC. They found that prolonged amygdala response and a functional disconnection between ventral and, additionally, dorsal – mPFC regions, conceived to be a part of the neural mechanisms underlying emotional dysregulation in BPD patients. Based on these results, our findings support the hypothesis, that BPD patients have difficulty regulating frightening emotions top-down using cognitive control processes (Buchheim et al., 2016). Additionally, they could be linked to findings on structural alterations in white-matter tracts connected to paralimbic and prefrontal brain regions in the same sample (Lischke et al., 2015, 2017).

#### Personalized Attachment Material and Unresolved Attachment Representation

The fMRI paradigm by Buchheim et al. (2012), which was originally employed for studying depressive patients, also allowed in the present study a robust investigation of the neural signature of personalized attachment material versus neutral textual material in a group of BPD patients and HC. On a behavioral level, the BPD patients as expected showed increased negative emotional valence and autobiographic relevance of all personalized sentences extracted from the AAP stories. Corresponding to the high impact of the own attachment experiences (Zanarini et al., 2016), the patients demonstrated enhanced fMRI-activation of the amygdala compared to HC when presented with personalized but not neutral sentences combined with monadic AAP pictures. Buchheim et al. (2012) also detected psychotherapy-induced changes that affected depressive patients but not controls, only in the appraisal of personalized – but not the neutral – attachment material, localized in the left amygdala extending laterally into the anterior hippocampus and toward the middle temporal gyrus. In a meta-analysis of Bakermans-Kranenburg and van IJzendoorn (2009) depressive patients compared with healthy groups showed a higher percentage of organized insecure attachment representations, whereas BPD patients showed a higher percentage of unresolved attachment representations. We may conclude that insecure attachment, in general, may reflect increased levels of amygdala activation in response to personalized attachment statements as it activates the attachment system via representation.

Additionally, only BPD patients in our study showed a positive association between number of unresolved rated AAP pictures and aMCC activation during monadic personalized sentence presentation. The aMCC plays an important role in the integration of neural circuitry for affect regulation due to connection to both emotional-limbic structures including the amygdala and the prefrontal cortex (Stevens et al., 2011).

Several areas showing increased fMRI-activation when BPD patients observe monadic pictures in comparison to HCs, had been related to pain processing. Physical and emotional pain share a common neuroanatomical basis (Eisenberger et al., 2003). A comprehensive literature review led Vogt (2005) to specify this overlap as related to three brain subsystems relevant for pain. These include fear-avoidance in the aMCC [for emotional processing literature predominantly termed the dorsal part of the anterior cingulate cortex (dACC)], unpleasantness in the posterior part of ACC (pACC), and skeletomotor orientation of the body in response to a noxious stimulus in the posterior part of the midcingulate cortex (pMCC) and dorsal part of the posterior cingulate cortex (dPCC). Vogt (2005) specified that the fear signal in aMCC may be more closely associated with predicting behavioral outcomes than sensory affect *per se*. Fear and pain representation overlap in the aMCC and support a more general role of this region for avoidance behavior.

Consequently, the current result could be interpreted as a neural signature of increasing social pain for BPD patients with unresolved attachment representation when they have to consciously face autobiographical abandonment themes.

### Limitations

The study results must be considered in light of several limitations. First, all attachment interviews were conducted before fMRI scanning to determine the participants’ attachment classification group and obtain the narrative material required to develop the personalized sentences for the fMRI procedure. Repeating core sentences from their AAPs during fMRI scanning cannot exclude the potential confounding effects of memory. However, patients did not report a recognition effect when asked after the fMRI experiment. Second, the content of personalized sentences was not analogous across the two groups (BPD, HC). This would have been impossible if we were to claim, as we did, that the material was personalized. Indeed, HC statements were less traumatic than the BPD statements, and HC rated the personalized sentences as less personally relevant and less emotional negative than the BPD patients. Third, all participants were female, so we cannot generalize our results to male individuals. Fourth, 35% of our patients were taking antidepressant medications, which might have had an additional effect on fMRI activation. However, subgroup analyses revealed no differences. Fifth, we had a wide range of patients with comorbidity of Posttraumatic Stress Disorder (PTSD; 39%); however, no differences in the fMRI activation patterns could be found between patients with/without PTSD. Sixth, we did not include a psychophysiological control such as heart rate during scanning, so we could not independently ensure our hypothesis of high arousal with thalamic hyperactivation in BPD patients while viewing personalized, monadic AAP pictures. Seventh, we had a multi-factorial design and calculated a number of different activation contrasts. In this context, we did not waive multiple testing and included the risk of alpha error cumulation. Therefore, all results were hedged by ROI- and FWE correction. Moreover, future studies should investigate the functional connectivity between relevant neural areas in activation of the internal working model of attachment. In spite of these limitations, this is the first study in BPD patients and matched HC exploring the neural signature of attachment representation comparing the impact of different attachment-relevant stimuli.

## Conclusion

Our results show a complex signature of social pain processing in BPD patients in response to attachment-relevant stimuli representing the state of being left alone and potentially abandoned. The present study extends previous functional characterizations of an attachment-relevant network. We specifically identified regions that comprise mentalization-related processes and self-awareness (pSTS, mPFC, and temporal poles), regions processing the perception and empathy of pain and fear (aMCC, amygdala, and anterior insula), and regions involved in conflict monitoring, cognitive control, reaction inhibition (aMCC and vmPFC), and arousal (thalamus). Some patterns confirm previous studies (Vogt, 2005; Buchheim et al., 2008) that showed the aMCC region seems to play an important role in activation of the internal working model of attachment in BPD patients. Moreover, our results support the hypothesis of hypermentalization in response to attachment distress as a core feature of social-cognitive impairment in the context of BPD (Fonagy et al., 2017; Somma et al., 2019), associated with common treatment implications across different therapeutic orientations (Choi-Kain et al., 2017). Dialectical Behavioral Therapy (DBT; Linehan et al., 1991) proposes that individuals with BPD can become more effective in managing their sensitivities and interactions with others through acquisition of skills that enhance mindfulness and enable them to better tolerate distress, regulate their emotions, and manage relationships. An RCT study on Transference Focused Psychotherapy (TFP; Clarkin et al., 2006) demonstrated for the first time a significant shift from BPD patients with unresolved trauma to organized attachment representations after 1 year of treatment (Buchheim et al., 2017a). Here, TFP was superior in revealing changes compared to Therapy as Usual. The highly structured interactive and emotionally intensive stance of the TFP therapist reflected an appropriate setting reflecting on adverse attachment relationships. Such intrapsychic changes can be considered as relevant for long-term treatment benefits (Buchheim et al., 2017a). In terms of deriving treatment implications, our present results support the significance of attachment trauma in patients with BPD. In that regard further studies might examine the effectiveness of Dialectical Behavior Therapy for Posttraumatic Stress Disorder (DBT-PTSD) for patients with PTSD after childhood sexual abuse in the presence of severe co-occurring psychopathology such as BPD (Bohus et al., 2013).

## Data Availability Statement

The raw data supporting the conclusions of this article will be made available by the authors, without undue reservation.

## Ethics Statement

The studies involving human participants were reviewed and approved by the Ethical Commission of the University of Greifswald (BB 136/10). The patients/participants provided their written informed consent to participate in this study.

## Author Contributions

DB, ML, and AB designed the study, interpreted the data, and wrote the manuscript. DB, RM, MD, and ML recruited the subjects and were responsible for data acquisition. DB and ML performed the statistical analysis. All authors read and approved the final manuscript.

## Conflict of Interest

The authors declare that the research was conducted in the absence of any commercial or financial relationships that could be construed as a potential conflict of interest.

## Publisher’s Note

All claims expressed in this article are solely those of the authors and do not necessarily represent those of their affiliated organizations, or those of the publisher, the editors and the reviewers. Any product that may be evaluated in this article, or claim that may be made by its manufacturer, is not guaranteed or endorsed by the publisher.

## Abbreviations

AAP: adult attachment projective picture system
BPD: borderline personality disorder
HCs: healthy control subjects
DBT: dialectical behavioral therapy
fMRI: functional magnet resonance imaging
R: resolved attachment
U: unresolved attachment
FEW: familywise error rate
ROI: region of interest
ACC: anterior cingulate cortex
MCC: medial cingulate cortex
PCC: posterior cingulate cortex
PFC: prefrontal cortex
STS: superior temporal cortex.
