# Supplementary material for: Neural Correlates of Attachment Representation in Patients With Borderline Personality Disorder Using a Personalized Functional Magnet Resonance Imaging Task
Source: Front Hum Neurosci. 2022 Feb 24;16:810417. doi: 10.3389/fnhum.2022.810417 (PMC8908102; doi:10.3389/fnhum.2022.810417)
Supplement: Supplementary file 1 [file Data_Sheet_1.PDF]

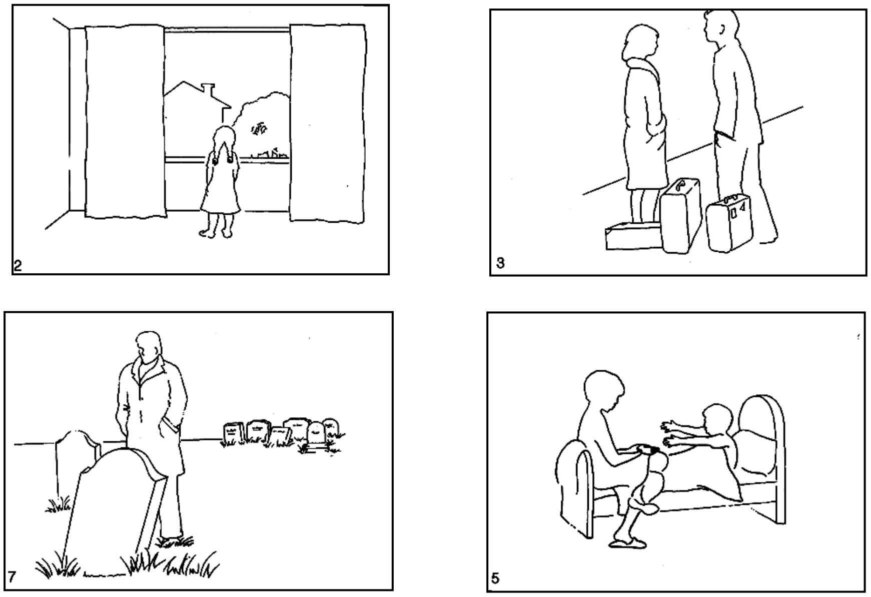

Monadic Pictures Dyadic Pictures

Supplementary Material 1. Examples of Pictures of the Adult Attachment Projective Picture System (AAP) (George & West, 2012; © all rights reserved)

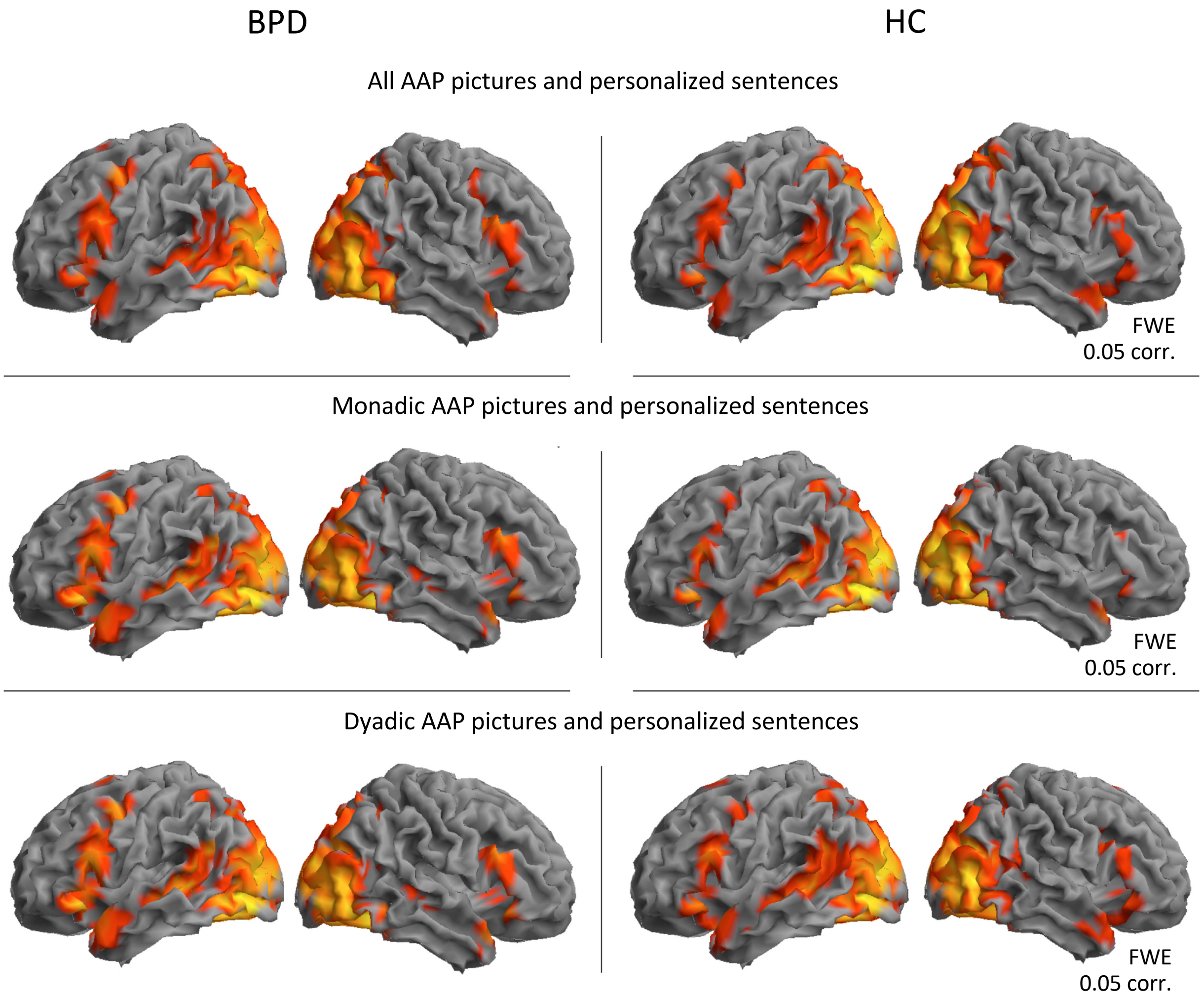

Supplementary Material 2. Overview on main effects (BPD vs. HC). All participant groups showed bilateral a fronto-temporal and occipital fMRI-activation ($p < 0.05$, FWE whole brain corrected)
